# Hepcidin and Anemia: A Tight Relationship

**DOI:** 10.3389/fphys.2019.01294

**Published:** 2019-10-09

**Authors:** Alessia Pagani, Antonella Nai, Laura Silvestri, Clara Camaschella

**Affiliations:** ^1^Division of Genetics and Cell Biology, San Raffaele Scientific Institute, Milan, Italy; ^2^Vita-Salute San Raffaele University, Milan, Italy

**Keywords:** anemia, iron, hepcidin, erythropoiesis, inflammation

## Abstract

Hepcidin, the master regulator of systemic iron homeostasis, tightly influences erythrocyte production. High hepcidin levels block intestinal iron absorption and macrophage iron recycling, causing iron restricted erythropoiesis and anemia. Low hepcidin levels favor bone marrow iron supply for hemoglobin synthesis and red blood cells production. Expanded erythropoiesis, as after hemorrhage or erythropoietin treatment, blocks hepcidin through an acute reduction of transferrin saturation and the release of the erythroblast hormone and hepcidin inhibitor erythroferrone. Quantitatively reduced erythropoiesis, limiting iron consumption, increases transferrin saturation and stimulates hepcidin transcription. Deregulation of hepcidin synthesis is associated with anemia in three conditions: iron refractory iron deficiency anemia (IRIDA), the common anemia of acute and chronic inflammatory disorders, and the extremely rare hepcidin-producing adenomas that may develop in the liver of children with an inborn error of glucose metabolism. Inappropriately high levels of hepcidin cause iron-restricted or even iron-deficient erythropoiesis in all these conditions. Patients with IRIDA or anemia of inflammation do not respond to oral iron supplementation and show a delayed or partial response to intravenous iron. In hepcidin-producing adenomas, anemia is reverted by surgery. Other hepcidin-related anemias are the “iron loading anemias” characterized by ineffective erythropoiesis and hepcidin suppression. This group of anemias includes thalassemia syndromes, congenital dyserythropoietic anemias, congenital sideroblastic anemias, and some forms of hemolytic anemias as pyruvate kinase deficiency. The paradigm is non-transfusion-dependent thalassemia where the release of erythroferrone from the expanded pool of immature erythroid cells results in hepcidin suppression and secondary iron overload that in turn worsens ineffective erythropoiesis and anemia. In thalassemia murine models, approaches that induce iron restriction ameliorate both anemia and the iron phenotype. Manipulations of hepcidin might benefit all the above-described anemias. Compounds that antagonize hepcidin or its effect may be useful in inflammation and IRIDA, while hepcidin agonists may improve ineffective erythropoiesis. Correcting ineffective erythropoiesis in animal models ameliorates not only anemia but also iron homeostasis by reducing hepcidin inhibition. Some targeted approaches are now in clinical trials: hopefully they will result in novel treatments for a variety of anemias.

## Introduction

Anemia is one of the most common disorders worldwide and anemia due to iron deficiency is the prevalent form according to multiple analyses (review in Camaschella, 2019). This type of anemia results from the total body iron deficiency and the inability to supply the large amount of iron that the bone marrow consumes to produce an adequate number of red blood cells in order to maintain tissue oxygenation.

The iron availability is controlled by the liver peptide hormone hepcidin. The body iron increase causes the production of hepcidin, which is released in the circulation and acts on its receptor ferroportin, a transmembrane iron exporter protein highly expressed on enterocyte, macrophages, and hepatocytes. Hepcidin reduces the iron entry to plasma from absorptive duodenal cells and iron recycling macrophages by blocking iron export (Aschemeyer et al., 2018) and by degrading the iron exporter ferroportin (Nemeth et al., 2004). By regulating plasma iron and systemic iron homeostasis, the hepcidin/ferroportin axis strongly affects erythropoiesis, hence the possible development of anemia.

## The Iron-Erythropoiesis Connection

The process of red blood cells production consumes approximately 80% of circulating iron for hemoglobin synthesis of maturing erythroblasts. Most iron (20–25 mg/daily) is recycled by macrophages, while a limited amount (1–2 mg daily) derives from intestinal absorption. The kidney hormone erythropoietin (EPO) controls the proliferation of erythroid progenitors, especially of CFU-e and at a lower degree of BFU-e, and the early phase of terminal erythropoiesis, while iron needs are increased in the late differentiation stages from proerythroblasts to reticulocyte, for the synthesis of heme incorporated into hemoglobin (Muckenthaler et al., 2017).

Hepcidin regulation requires a crosstalk between liver endothelial sinusoidal cells (LSEC) that produce the bone morphogenetic proteins (BMPs) to activate the BMP-SMAD pathway and hepatocytes that produce and release hepcidin (Babitt et al., 2006; Rausa et al., 2015). BMP6 and BMP2 are the most important BMPs that upregulate hepcidin, while BMP6 expression is iron dependent (Andriopoulos et al., 2009; Meynard et al., 2009) BMP2 appears less iron-responsive (Canali et al., 2017; Koch et al., 2017).

Hepcidin levels are low in absolute iron deficiency and iron deficiency anemia. In these conditions, the iron stores are exhausted and the BMP-SMAD signaling is switched off at multiple levels. First, BMP6 expression is suppressed; next, the activity of TMPRSS6, a protease that cleaves the BMP co-receptor hemojuvelin (Silvestri et al., 2008), is strongly increased (Lakhal et al., 2011); and third, histone deacetylase3 (HDAC3) suppresses the hepcidin locus (Pasricha et al., 2017). In conditions of iron deficiency, the reduction of hepcidin production is an adaptation mechanism that facilitates dietary and pharmacological iron absorption (Camaschella and Pagani, 2018).

When anemia is severe, the coexisting hypoxia stimulates erythropoiesis through increased kidney synthesis and release of EPO. This leads to suppression of hepcidin transcription by erythroferrone (ERFE), an EPO target gene produced by erythroblasts (Kautz et al., 2014), by molecules (e.g., PDGF-BB) released by other tissues (Sonnweber et al., 2014), and likely by soluble components of transferrin receptors (TFR), sTFR1 (Beguin, 2003), and sTFR2 (Pagani et al., 2015). The final aim is to supply enough iron for the needs of an expanded erythropoiesis.

## Anemias With Abnormal Hepcidin Levels

**Figure 1:**
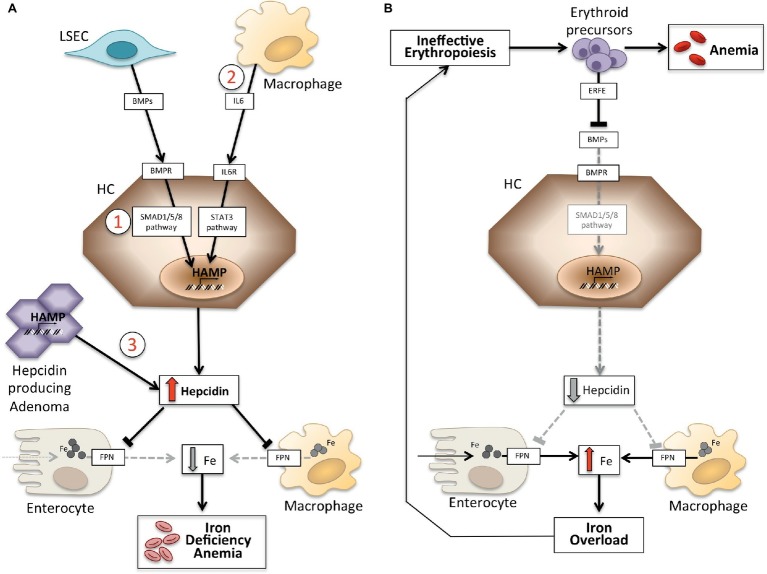
Schematic representation of mechanisms of anemias with high (left panel) and low hepcidin (right panel). Panel **(A)**. Molecular pathogenesis of anemia associated with high hepcidin levels. LSEC, liver sinusoidal endothelial cells producing bone morphogenetic proteins (BMPs); BMPRs, BMP receptors; IL6, interleukin 6; HC, hepatocytes; HAMP, hepcidin gene. Fe, iron; FPN, ferroportin; 1, IRIDA; 2, Anemia of inflammation; 3, hepcidin producing adenoma. Panel **(B)**. Molecular pathogenesis of hepcidin variation in anemias due to ineffective erythropoiesis. ERFE, erythroferrone sequestering BMPs. Other mechanisms inhibiting hepcidin in this type of anemia, as decrease of transferrin saturation and hypoxia, are not shown. See text for details.

Anemias may be classified on the basis of hepcidin levels as anemias with high and low hepcidin. It is intuitive that persistently high hepcidin levels, by blocking iron absorption, cause iron deficiency anemia because of decreased iron supply to erythropoiesis. Conversely, ineffective erythropoiesis characterizes the so-called *iron-loading anemias* that have low hepcidin levels and iron overload. These two groups of anemias are the outcome of opposite pathophysiology mechanisms (Figure 1). In the first group, anemia is due to the inhibitory effect exerted by hepcidin on iron absorption and recycling that leads to systemic iron deficiency; in the second group, anemia is due to hepcidin suppression by an expanded abnormal erythropoiesis (Camaschella and Nai, 2016).

### Anemia Associated With High Hepcidin Levels

This group includes two inherited rare disorders (iron refractory iron deficiency anemia and hepcidin-producing adenomas in an inborn error of glucose metabolism) and an acquired common condition: anemia of inflammation (Table 1).

**Table 1:** Anemias classified according to hepcidin levels.

**High-hepcidin anemias**		
Hereditary	OMIM n.	Prevalence
Iron refractory iron deficiency anemia (IRIDA)	#206200	Rare
Hepcidin-producing adenomas^*^	#232200	Rare
Acquired		
Anemia of acute inflammation		Common^**^
Anemia of chronic inflammation (anemia of chronic disease)		Common
**Low-hepcidin anemias**		
Hereditary – iron loading anemias	OMIM n.	
β-thalassemia	**#**613985	Common^***^
Congenital dyserythropoietic anemia	**#**224100	Rare
Sideroblastic anemias	**#**300751	Rare
Acquired		
Low risk MDS with ringed sideroblasts		Rare

*OMIM, online Mendelian Inheritance In Man; MDS, myelodysplastic syndromes*.

* *Described in glycogen-storage-disease 1a*.

** *In hospitalized patients and in intensive care units*.

*** *In people of Mediterranean or southern-east Asian origin*.

### Iron Refractory Iron Deficiency Anemia

Iron refractory iron deficiency anemia (IRIDA) is a rare recessive disorder characterized by hypochromic microcytic anemia, low transferrin saturation, and inappropriately normal/high hepcidin levels. It is caused by mutations of *TMPRSS6* (Finberg et al., 2008), a gene that encodes the type II serine protease, matriptase-2 (Du et al., 2008). Mutations of *TMPRSS6* are spread along the gene and may affect different domains especially the catalytic domain (De Falco et al., 2014). This transmembrane protease, highly expressed in the liver, inhibits hepcidin transcription by cleaving the cell surface BMP co-receptor hemojuvelin, thus attenuating the BMP signaling and hepcidin synthesis (Silvestri et al., 2008). TMPRSS6 function is essential in iron deficiency to allow the compensatory mechanism of increased iron absorption.

IRIDA is present since birth and usually diagnosed in childhood. Compared with classic iron deficiency, iron parameters are atypical and raise the suspicion of the disease. The percent saturation of transferrin is strongly reduced (less than 10%) as in other forms of iron deficiency; however, at variance with iron deficiency, levels of serum ferritin are normal/increased (Camaschella, 2013; De Falco et al., 2013). This reflects an increased ferritin accumulation in macrophages, due to the high hepcidin levels that induce store iron sequestration.

None of the tests proposed for IRIDA diagnosis covers 100% of the cases. The genetic test identifies that *TMPRSS6* mutations, that in some cases (non-sense, frame-shift, and splicing mutations), are clearly causal. In other cases, as for previously unreported missense mutations, functional studies are needed to demonstrate causality (Silvestri et al., 2013). However, these tests are scarcely available. Serum hepcidin levels are usually increased/normal, independently of iron deficiency, and consistent with high/normal ferritin. It is important to exclude inflammation by concomitantly dosing C-reactive protein.

Some patients with a phenotype of refractory iron deficiency have been reported to have a single *TMPRSS6* mutated allele; here, the debate is whether they should be considered IRIDA or not. A spectrum of conditions can be envisaged ranging from classic severe IRIDA due to homozygous or compound heterozygous *TMPRSS6* mutations to increased susceptibility to iron deficiency conferred by single mutations/polymorphic changes. One approach proposed to predict classic IRIDA is hepcidin normalization on other iron parameters, as ratios transferrin saturation (Tsat)/log hepcidin or Tsat/log Ferritin (Donker et al., 2016). According to other authors, most patients with a severe IRIDA phenotype have biallelic *TMPRSS6* mutations and, when unidentified, the second allele may be genetically occult (Heeney et al., 2018). In general terms, subjects with a single allele have a milder phenotype than those with two mutations and respond better to iron treatment (Donker et al., 2016). Interestingly, several *TMPRSS6* SNPs have been shown to provide susceptibility to iron deficiency in some populations (An et al., 2012) and in blood donors (Sorensen et al., 2019).

A digenic inheritance has been reported in a 5-year-old female originally found to have an atypical IRIDA genotype with one *TMPRSS6* (I212T) causal and one (R271Q) silent mutation (De Falco et al., 2014). She was later diagnosed *Fibrodysplasia ossificans progressiva* (FOP), a rare dominant disorder with ectopic bone formation in soft tissues due to mutated BMP type I receptor gene *ACVR1*, encoding ALK2 (Shore et al., 2006). The pathological allele *ALK2^R258S^* is constitutively active since the mutation affects the glycine-serine-rich domain of the gene and renders the BMP/SMAD pathway overactive being unable to bind its specific inhibitor FKBP12 (Pagani et al., 2017).

This rare case is especially illustrative. First, since the ALK2 glycine-serine-rich domain interacts with FKBP12 and the mutation destabilizes the binding, it has revealed a previously unsuspected role for FKBP12 as a modulator of liver ALK2 and hepcidin (Colucci et al., 2017). Second, it has led to identify a link between activation of bone and liver BMP type I receptors. Third, the case strengthens the relevance of intact TMPRSS6 in controlling the hepatic BMP/SMAD signaling, since no IRIDA was identified among other FOP patients with the same *ACVR1* mutation and presumably normal *TMPRSS6* (Pagani et al., 2017). Finally, this case is consistent with the concept that *TMPRSS6* haploinsufficiency cannot cause classic IRIDA.

The optimal treatment of IRIDA is undefined. Oral iron is ineffective, since it is not absorbed. The addition of vitamin C allows sporadic response. Intravenous iron induces a partial response usually at a slower rate in comparison with patients with acquired iron deficiency. EPO is ineffective in classic cases (De Falco et al., 2013; Heeney and Finberg, 2014).

### Anemia of Hepcidin-Producing Adenomas

This is an extremely rare condition in adult patients affected by *glycogen storage disease 1a*, a recessive disorder due to deficiency of glucose-6 phosphatase, which catalyzes a reaction involved in both glycogenolysis and gluconeogenesis. A common dangerous disease symptom is hypoglycemia. The current treatment leads to prolonged survival of affected children up to adult age with the occurrence of several complications, such as anemia and liver adenomas. Anemia is microcytic and hypochromic, iron deficient, and refractory to oral iron treatment. Anemia reverted after surgical adenoma resection. Adenoma tissue was found positive for hepcidin mRNA, while normal surrounding tissue showed hepcidin suppression, as expected because of the ectopic uncontrolled hepcidin production (Weinstein et al., 2002). The hematological features of patients resemble those of IRIDA as they share high hepcidin levels as a common mechanism of anemia.

### Anemia of Inflammation

Anemia of inflammation (AI), previously known as anemia of chronic diseases, is a moderate normochromic-normocytic anemia that develops in conditions of systemic inflammation and immune activation. It occurs in several common disorders, including chronic infections, autoimmune diseases, advanced cancer, chronic kidney disease, congestive heart failure, chronic obstructive pulmonary disease, anemia of the elderly (at least partly), and graft versus host disease. AI is one of the most common anemias worldwide and the most frequent anemia in hospitalized patients. Acute inflammation contributes to the severity of anemia in intensive care units. Molecular mechanisms underlying AI are multiple and complex. Overproduction of cytokines such as IL1-β, TNF-α, and IL-6 by macrophages and INF-γ by lymphocytes blunts EPO production, impairs the erythropoiesis response, increases hepcidin levels, and may activate erythrophagocytosis, especially in the acute forms (Weiss and Goodnough, 2005; Ganz, 2019).

Hepcidin is activated by IL-6 through IL-6 receptor (IL-6R) and JAK2-STAT3 signaling. Full hepcidin activation requires an active BMP-SMAD pathway because inactivation of BMP signaling decreases hepcidin in animal models of inflammation (Theurl et al., 2011). The deregulation of systemic iron homeostasis causes macrophage iron sequestration and reduced absorption and recycling that leads to low saturation of transferrin and iron restriction of erythropoiesis and other tissues.

Traditional treatment of AI is based on reversibility/control of the underlying disease, whenever possible. If the disease is untreatable and anemia is mild, a careful evaluation of risks-benefits is needed to avoid side effects of any treatment. Pathophysiology-based treatments are limited to erythropoietin-like compounds and iron. The use of erythropoiesis stimulating agents (ESA) suppresses hepcidin by inducing erythropoiesis expansion. This approach is widely used in patients with chronic kidney disease, low-risk myelodysplastic syndromes, and cancer undergoing chemotherapy. However, a careful clinical control is necessary because high doses have cardiovascular side effects. The administration of intravenous iron may relieve iron restriction, caused by ESA-dependent expansion of erythropoiesis. Oral iron is usually ineffective since the high hepcidin levels counteract its intestinal absorption. Inhibitors of prolyl hydroxylase (hypoxia inducible factor, HIF stabilizers) are experimental in chronic kidney disease, to the aim of increasing endogenous EPO. Chronic treatment with red blood cells transfusions is not recommended because of transient effect and adverse reactions; it is limited to severe refractory anemia (Camaschella, 2019; Weiss et al., 2019).

### Anemias Associated With Low Hepcidin Levels

Ineffective erythropoiesis and low or inappropriately normal hepcidin levels, with consequent iron overload, are typical features of the *“iron-loading anemias*.*”* The prototype is β-thalassemia, a genetic recessive disease due to β-globin gene mutations that cause anemia and excess α-globin chain production. The latter precipitates as hemichromes in the bone marrow, damaging maturing erythroid precursors and leading to ineffective erythropoiesis. This occurs in non-transfusion-dependent thalassemia or thalassemia intermedia, whose erythropoiesis is characterized by the prevalence of immature cells that release erythroferrone to inhibit liver hepcidin expression. Hepcidin levels are usually greater in transfusion-dependent thalassemia, where endogenous ineffective erythropoiesis is at least partially suppressed by transfusions (Camaschella and Nai, 2016).

Hepcidin suppression is mediated by the increased cytokine erythroferrone (ERFE), a member of the TNF-α family encoded by *ERFE* gene, synthesized by erythroblasts upon EPO stimulation (Kautz et al., 2014). ERFE is released into the circulation and sequesters BMPs, especially BMP6 (Arezes et al., 2018), attenuating the hepcidin signaling in response to iron. In addition, an epigenetic suppression occurs at the hepcidin locus by histone deacetylase HDAC3 (Pasricha et al., 2017). When anemia causes hypoxia, other mediators such as PDGF-BB (Sonnweber et al., 2014), which is released by different cell types, suppress hepcidin.

Hepcidin levels are decreased by a special mechanism in low-risk myelodysplasia with ringed sideroblasts, a clonal disorder due to mutations of the spliceosome gene *SF3B1.* Iron accumulates in mitochondria, leading to ineffective erythropoiesis and systemic iron overload. An abnormally spliced, elongated ERFE protein is more powerful than wild type ERFE in suppressing hepcidin (Bondu et al., 2019) and causing transfusion-independent iron loading.

## Targeted Therapies for Hepcidin-Related Anemias

The identification of molecular mechanisms responsible of the previously discussed anemias has stimulated research in developing targeted therapies to replace current symptomatic treatment (Sebastiani et al., 2016; Crielaard et al., 2017). Approaches differ according to the type of anemia and the aim of decreasing or increasing hepcidin levels or their effects (Table 2).

**Table 2:** Experimental therapies targeting the hepcidin-ferroportin axis.

	Mechanism	Compounds
**Compounds that decrease hepcidin or increase ferroportin function**		
Class I	Reduction of the signaling pathway stimulating hepcidin	Anti IL6-R, anti IL-6 Anti-BMP6 MoAb^*^ BMPR inhibitors Anti-HJV MoAb Non anticoagulant heparins
Class II	Hepcidin binders	Anti-HAMP MoAb Oligonucelotides aptamers
Class III	Interfering with hepcidin-FPN interaction	Anti-FPN MoAb, GDP
**Compounds that increase hepcidin or decrease ferroportin function**		
Class I	Hepcidin mimics	Hepcidin analogues^*^ Minihepcidin
Class II	Activating hepcidin	BMPs (preclinical studies)
	Blocking the hepcidin inhibitor	Anti-*TMPRSS6* (siRNA, ASO^*^)
	Blocking the hepcidin receptor	FPN Inhibitors^*^
Class III	Others	Human transferrin infusions
		Protoporphyrin IX (inhibition of HO)
		Bone marrow TFR2 inactivation

*BMPR, BMP receptor; HAMP, hepcidin gene; HJV, hemojuvelin; MoAb, monoclonal antibody; FPN, ferroportin; siRNA, small interfering RNA; ASO, antisense oligonucleotides; GDP, guanosine 5′ diphosphate; HO, heme oxygenase; TFR2, transferrin receptor 2*.

Compounds indicated by ^*^ are in clinical trials.

### Experimental Therapies to Decrease Hepcidin Levels/Increase Ferroportin Function

Except for hepcidin producing tumors, which have to be surgically removed, compounds that antagonize hepcidin or its effects may be useful in all anemias characterized by high hepcidin levels. Their main application would be in chronic inflammatory diseases in order to reverse hypoferremia and anemia. Several experimental therapies aimed at manipulating the hepcidin pathway and its function have been investigated in preclinical studies. Hepcidin antagonists are inhibitors of hepcidin synthesis/regulators (Ganz, 2019), hepcidin binders that block its function, and compounds that interfere with hepcidin-ferroportin interaction (Table 2). Some compounds are in clinical trials especially in chronic kidney disease (Sheetz et al., 2019). In IRIDA, manipulation of the hepcidin pathway has been proposed in preclinical studies with the use of anti-HJV MoAb (Kovac et al., 2016).

### Experimental Therapies to Increase Hepcidin Levels/Decrease Ferroportin Function

Increasing hepcidin levels may not only reduce iron overload but also partially control ineffective erythropoiesis in *iron loading anemias.* β-thalassemia is the most studied among these conditions (Casu et al., 2018; Gupta et al., 2018). Proposed drugs are hepcidin analogs (some in clinical trials), hepcidin modulators, especially TMPRSS6 inhibitors, or compounds that interfere with hepcidin-ferroportin interaction decreasing iron export (Table 2).

While compounds that increase hepcidin reduce ineffective erythropoiesis due to the vicious cycle between ineffective erythropoiesis and iron loading (Camaschella and Nai, 2016), drugs that favor erythroid precursor maturation, as the activin receptor IIB ligand trap, luspatercept, not only improve anemia but also ameliorate iron homeostasis by reducing hepcidin inhibition (Piga et al., 2019).

Some targeted approaches now in clinical trials will hopefully result in novel treatments for a variety of anemias.

## Conclusion

The spectacular advances in understanding the regulation of iron metabolism and hepcidin allowed a better understanding of erythropoiesis control, since together with erythropoietin iron is a fundamental factor for erythroid cells maturation. Conditions that lead to anemia can be associated with high and low hepcidin levels. In both instances, contrasting hepcidin deregulation may ameliorate/correct anemia in preclinical models, offering new tools that are already or will be soon clinically explored for the treatment of specific anemias.

## Author Contributions

AP drafted the paper. CC developed the final version. AN and LS contributed to writing and to critical review the manuscript. All the authors approved the final version.

### Conflict of Interest

CC is a consultant of Vifor Pharma, Celgene, and Novartis.

The remaining authors declare that the research was conducted in the absence of any commercial or financial relationships that could be construed as a potential conflict of interest.
